# Slow PP_i_ release enhances fidelity of the SARS-CoV-2 RNA dependent RNA polymerase

**DOI:** 10.1016/j.jbc.2026.111457

**Published:** 2026-04-17

**Authors:** Tyler L. Dangerfield, Ingrid Marko, Kenneth A. Johnson

**Affiliations:** Department of Molecular Biosciences, The University of Texas at Austin, Austin, Texas, USA

**Keywords:** mismatch incorporation, pre-steady-state kinetics, pyrophosphate release, replication fidelity, RNA-dependent RNA polymerase, SARS-CoV-2

## Abstract

Viral RNA-dependent RNA polymerases (RdRps) must balance replication speed with fidelity, preserving genome integrity while permitting enough variability for viral adaptation. The SARS-CoV-2 RdRp complex (non-structural protein 12/7/8) achieves this through the interplay of its intrinsic replication fidelity and a potential proofreading exonuclease complex (NSP10/14). Here, we comprehensively quantify the intrinsic fidelity of the SARS-CoV-2 RdRp through direct pre-steady-state kinetic analyses of nucleotide incorporation across all possible templating bases paired with incoming nucleotides. We discovered substantial variation in discrimination against mismatches ranging from one error in 10^3^ to 10^8^ (median of 10^5^). Crucially, our data reveal a slow pyrophosphate release step that significantly enhances fidelity by effectively introducing a kinetic checkpoint after nucleotide incorporation. The error rates we measured for the RdRp align closely with observed *in vivo* mutation rates, suggesting that the exonuclease complex may play a less critical role than previously assumed in correcting mistakes during polymerization. These insights advance our understanding of SARS-CoV-2 replication fidelity, and the role of various subcomplexes in genome maintenance and adaptation.

Viral polymerases must replicate their genomes with both speed and accuracy, preserving integrity while generating the diversity required for adaptation. Genetic variation allows viruses to effectively respond to selective pressures such as host immune defenses, antiviral therapeutics, and vaccines. SARS-CoV-2, the virus responsible for the COVID-19 pandemic, encodes an RNA-dependent RNA polymerase complex (NSP12/7/8) and an exonuclease complex (NSP10/14), which are believed to function together to modulate replication fidelity. The exonuclease is proposed to enhance fidelity by removing mismatched nucleotides (1, 2, 3). Our recent work raises questions about this role, as NSP10/14 displays catalytic activity magnitudes higher on perfectly base-paired RNA than on mismatched double-stranded RNA substrates (4). However, these measurements were performed with the exonuclease in isolation, not in complex with the RNA-dependent RNA polymerase (RdRp), which could govern selectivity of the exonuclease as seen with other polymerases (5, 6, 7).

Despite extensive characterization of the structures and biochemical properties of SARS-CoV-2 RdRp and exonuclease, fundamental questions persist. Specifically, what is the intrinsic replication fidelity of the NSP12/7/8 complex? Can this intrinsic fidelity alone explain the observed *in vivo* error rates of viral replication? Furthermore, what mutation spectrum arises during viral replication *in vivo*, and how does this spectrum correspond to the polymerase's misincorporation probabilities? Prior studies estimated a fidelity of only 10^−1^ to 10^−3^, corresponding to a discrimination of one error for every 10^1^ to 10^3^nt (8), but this work was flawed in that it relied on using manganese and steady-state analysis with a relatively dead enzyme, 200000-fold less active than our preparations (9).

In this report, we use single turnover kinetic analysis to directly measure nucleotide incorporation kinetics using synthetic RNA substrates encompassing all possible base pair combinations. The single turnover approach is essential because it bypasses the slow, rate-limiting enzyme dissociation step that dominates steady state measurements. Release of duplex RNA from the SARS-CoV-2 RdRp is 0.013 s^−1^ while polymerization occurs at 300—600 s^-1^ (9). By measuring incorporation on the timescale of a single turnover we bypass the slow RNA release step and therefore define the kinetic parameters (*k*_*cat*_/*K*_*m*_ and *k*_*cat*_) governing each incorporation reaction during processive synthesis. As we will show, for most misincorporation reactions, PP_i_ release is less than 0.013 s^-1^ and would limit steady-state turnover.

Notably, our methods provide the necessary dynamic range and temporal resolution to measure the concentration dependence of fast single nucleotide incorporation events. Quantitative analysis of the nucleotide concentration dependence of incorporation provides the probabilities for each of the 16 possible nucleotide incorporations from the specificity constants (*k*_*cat*_*/K*_*m*_). Our data reveal a wide spectrum of efficiencies in mismatch incorporation, with certain mismatches occurring over four orders of magnitude more frequently than others. Additionally, the kinetic data identify a prominent slow pyrophosphate (PP_i_) release mechanism, significantly enhancing fidelity against most mismatches. This slow PP_i_ release results in a reaction model where the catalytic turnover rate (*k*_*cat*_) and *K*_*m*_ are influenced by the reversibility of nucleotide incorporation and rate of slow PP_i_ release. The specificity constant, *k*_*cat*_/*K*_*m*_, is a function of all steps leading up to and including PP_i_ release.

## Results

**Figure 1:**
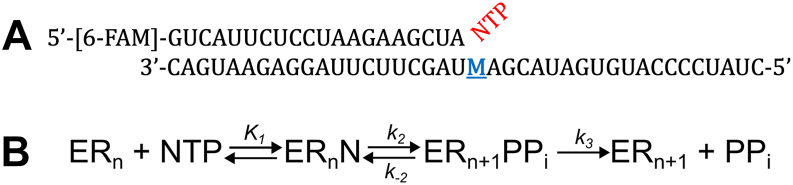
**RNA Substrate and Kinetic Scheme**. *A,* RNA substrate used in kinetics reactions. The templating base that is varied is shown in *blue* and labeled “M” while the incoming nucleotide is shown in *red*. *B,* kinetic scheme for misincorporation. The kinetic scheme used for data fitting is shown, where nucleotide binding and chemistry are modelled as reversible while the rate of pyrophosphate release is modeled as irreversible.

**Figure 2:**
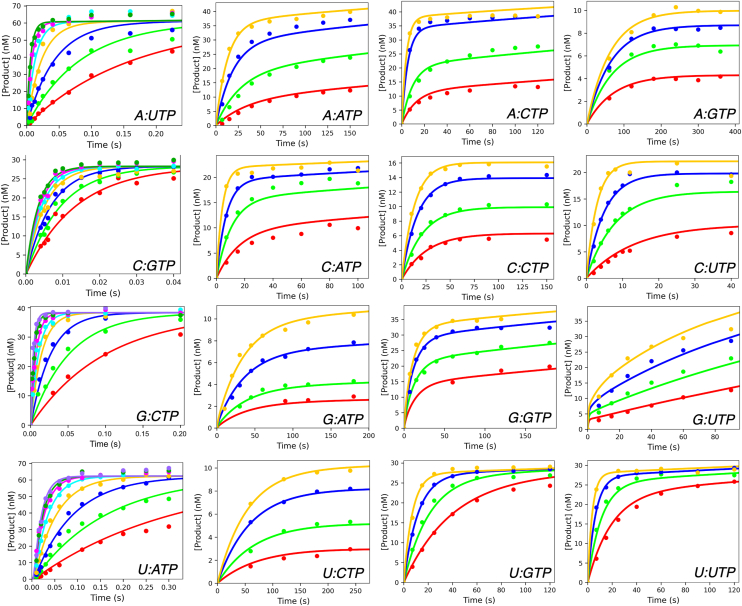
**Correct Incorporation and Misincorporation Kinetics**. For misincorporations, a solution of 2 μM SARS-CoV-2 NSP12/7/8, 6 μM NSP8, and 50 nM FAM-RNA was mixed with varying magnesium-nucleotide concentrations (between 100 and 4000 μM, depending on the mismatch) to start the reaction. For correct ATP and UTP incorporations, data were reproduced from our earlier study (9) and contained 2 μM SARS-CoV-2 NSP12/7/8, 6 μM NSP8 and 100 nM FAM RNA. For correct CTP and GTP incorporations, enzyme concentrations were the same as for ATP and UTP incorporations but had 50 nM FAM-RNA. Time points were quenched with EDTA and analyzed by capillary electrophoresis. Solid lines through the data represent best fits based on numerical integration of the rate equations to derive the rate constants listed in Table 1, from which key kinetic parameters were calculated as shown in Table 2 and plotted in Figure 3. Specific nucleotide concentrations used in each experiment are given in Table S1.

To directly quantify the intrinsic probability of each possible misincorporation by the SARS-CoV-2 RdRp, we performed presteady-state nucleotide incorporation experiments using RNA substrates containing all possible templating base and nucleoside triphosphate (NTP) combinations. The RdRp consists of four subunits: one copy of the catalytic subunit NSP12 and accessory proteins: one copy of NSP7 and two copies of NSP8. We use a shorthand term M:NTP to denote each pair, where M is the templating base and NTP is the incoming nucleoside triphosphate (Fig. 1). Polymerase and duplex RNA with a 5′-fluorescently labeled primer were pre-equilibrated for 30 min. Reactions were initiated by adding varying NTP concentrations in Mg^2+^-containing buffer using a rapid-quench flow. The resulting time dependence of product formation (Fig. 2) yielded both an observed rate and amplitude, each of which contains important mechanistic information (10). In particular, the amplitude reflects the fraction of bound RdRp-dsRNA complex that proceeds through the chemistry step. Fitting the data to a single exponential function ($y=a_{0}+a_{1}*\left(1-e^{-b1t}\right))$ yields an amplitude (*a*_*1*_) that defines the magnitude of the exponential phase, while the observed rate (*b*_*1*_) corresponds to the eigenvalue that is itself a function of the underlying microscopic rate constants.

Using conventional equation-based data fitting, a family of traces collected at seven different nucleotide concentrations (*e.g.*, the correct incorporation reactions in (Fig. 2)) would require 21 independent parameters. Interpretating such data would require separate analysis of the concentration dependence of both the rate and amplitude terms (10, 11). Traditional data fitting is limited because it treats rates and amplitudes as independent parameters, overlooking the inherent relationships between them. This inflates uncertainties and precludes direct derivation of intrinsic rate constants for multi-step reactions. In contrast, fitting data globally based on numerical integration of the rate equations enables quantitative analysis of the entire family of curves, yielding intrinsic rate constants directly (10). Fitting rates and amplitudes simultaneously as a function of nucleotide concentration defines the rate constants for a minimal model with 2 to 4 rate constants, depending on the kinetics (Fig. 1). This approach provides an accurate assessment of the reaction mechanism and rate constants without adding extra parameters. Accordingly, rather than attempting to interpret the results using equations that rely on necessary approximations, we fit the data directly to a minimal model that is both sufficient and supported by the experimental measurements. Here we provide a logical explanation of the observed reaction rates and amplitudes supported by our quantitative analysis.

Surprisingly, the amplitude of nearly all misincorporation reactions showed a dependence on nucleotide concentrations, a feature indicative of a slow PP_i_ release mechanism and reversible chemistry (11, 12, 13). In contrast, when PP_i_ release is fast, as is typical for correct incorporation reactions, the irreversible PP_i_ release step drives the polymerization reaction to completion so that the amplitude is independent of nucleotide concentration. As shown by direct measurement in prior studies (11, 12, 13), when PP_i_ release is slow, the chemical reaction comes to equilibrium and is thermodynamically linked to nucleotide binding. The minimal model to account for the data is shown in Figure 1. In this scheme, the equilibrium constant for ground state nucleotide binding (*K*_*1*_), is modelled as a rapid equilibrium step where *k*_*-1*_ >> *k*_*2*_, so the data only define the ratio of the on and off rate constants (*K*_*1*_=*k*_*1*_/*k*_*-1*_), but not either rate constant individually. Other key features of this model are the reversible chemistry step (*k*_*2*_ and *k*_*-2*_) and the largely irreversible, slow PP_i_ release (*k*_*3*_). Under the conditions of our assay, the concentration of free PP_i_ remained very low, justifying the irreversible PP_i_ release approximation. The time dependence of total product formation (denoted in our model as ER_n+1_PP_i_ plus ER_n+1_) is a function of all four constants and the nucleotide concentration.

To determine the rate constants giving rise to observed kinetics of incorporation, we globally fit the data for each misincorporation reaction using KinTek Explorer simulation software (14). This allowed analysis of the data without simplifying approximations, accounting for the nucleotide concentration dependence of the rate and amplitude of each curve and provided estimates for each of the rate constants governing incorporation. Confidence contour analysis with the FitSpace function (15) provided a crucial analysis to quantify the extent to which each parameter was constrained by the data. For correct nucleotide incorporations and for the U:guanosine triphosphate (GTP) misincorporation, PP_i_ release was fast, *k*_*3*_ >> *k*_*2*_. With no information to define *k*_*-2*_, the scheme collapses to a 2-step model. For all other misincorporations, *k*_*3*_ << *k*_*2*_, allowing the chemistry step to come to equilibrium, so the data define *k*_*2*_ and *k*_*-2*_. Rate constants for all incorporation reactions are summarized in Table 1. We expect that the chemistry step for correct incorporation may also be reversible, but rapid PP_i_ release eliminates the amplitude dependence of the reaction and precludes determination of *k*_*-2*_, and the value of *k*_-2_ does not influence estimates of *k*_*cat*_*/K*_*m*_.

**Table 1:** Rate constants for all possible base-pair combinations

Templating Base	NTP	*1/K*_*1*_ (μM)	*k*_*2*_ (s^-1^)	*k*_*-2*_ (s^-1^)	*k*_*3*_ (s^-1^)
A	ATP	33,000 (>10,800)	0.94 (>0.33)	0.019 (0.013–0.027)	0.0034 (<0.0073)
	CTP	1730 (1,30–2500)	0.35 (0.28–0.47)	0.057 (0.046–0.074)	0.0036 (0.0016–0.0059)
	GTP	4900 (3900–6800)	0.0072 (0.006–0.0092)	0.0128 (0.011–0.015)	< 0.0001
	UTP	130 (120–140)	310 (290–330)	[0]^a^	[10,000]^a^
C	ATP	7220 (>2400)	1.80 (>0.65)	0.033 (0.0200–0.0430)	0.0051 (0.002–0.008)
	CTP	13,100 (8600–26,000)	0.26 (0.18–0.47)	0.029 (0.027–0.032)	< 0.0005
	GTP	86 (66–110)	420 (360–480)	[0]^a^	[10,000]^a^
	UTP	7200 (>2100)	2.5 (>0.8)	0.045 (0.034–0.065)	< 0.001
G	ATP	2,90 (1800–4400)	0.034 (0.026–0.056)	0.022 (0.020–0.026)	< 0.001
	CTP	59 (49–69)	600 (500–700)	[0]^a^	[10,000]^a^
	GTP	1500 (970–3000)	0.41 (0.29–0.72)	0.054 (0.040–0.070)	0.0026 (0.0010–0.0040)
	UTP	560 (290–640)	0.12 (0.08–0.15)	0.038 (0.019–0.086)	0.009 (0.0017–0.018)
U	ATP	320 (270–370)	240 (210–270)	[0]^a^	[10,000]^a^
	CTP	5800 (4800–7500)	0.02 (0.016–0.024)	0.012 (0.010–0.013)	< 0.00001
	GTP	4100 (2400–10,000)	0.43 (0.28–0.97)	[0]^a^	[1000]^a^
	UTP	7000 (>4200)	1.2 (>0.76)	0.009 (0.0058–0.011)	< 0.011

a Parameters in brackets were locked during the fitting.

For correct incorporation reactions, the apparent ground-state nucleotide dissociation constant (1/*K*_*1*_) varied over a relatively small range, with extremes of 60 μM and 320 μM observed for G:cytidine triphosphate (CTP) and U:ATP incorporation, respectively. Values for the rate of catalysis, *k*_*2*_, varied from 240 to 600 s^-1^, extremes observed for U:ATP and G:CTP, respectively. Interestingly, the higher value of *K*_*d*_ (1/*K*_*1*_) for U:ATP is commensurate with the higher concentration of ATP in a cell (16). For misincorporation reactions, values of 1/*K*_*1*_ varied over a much larger range. At the extremes, 1/*K*_*1*_ for A:ATP misincorporation was 33 mM, while 1/*K*_*1*_ for G:GTP misincorporation was only 730 μM. Values for *k*_*2*_ ranged from 0.0072 s^-1^ for A:GTP misincorporation to 2.48 s^-1^ for C:UTP misincorporation. Values for *k*_*-2*_ ranged from 0 (not measurable) to 0.064. Finally, for all misincorporation reactions except for U:GTP, *k*_*3*_, the rate of PP_i_ release was very slow, with a maximum rate of 0.009 s^-1^.

**Figure 3:**
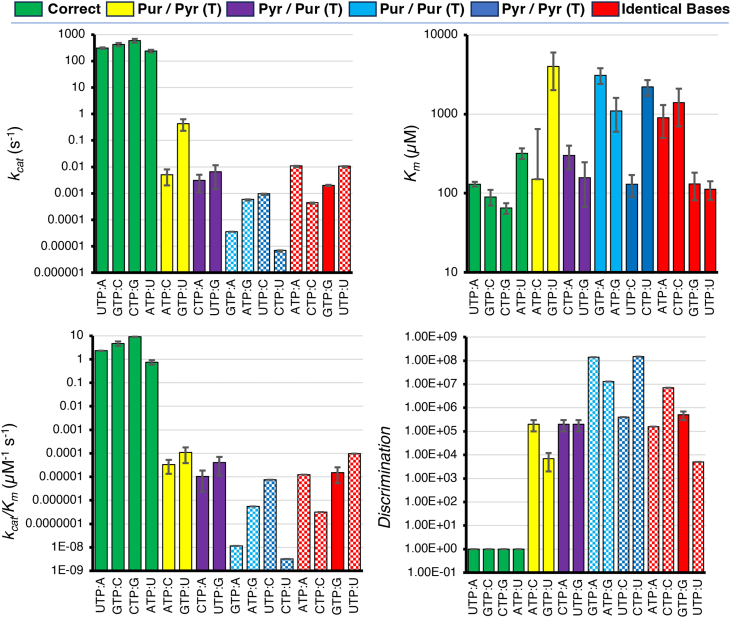
**Key Kinetic Parameters for Each Misincorporation Reaction**. Plots of the key kinetic parameters *k*_*cat*_, *K*_*m*_, *k*_*cat*_/*K*_*m*_, and discrimination (*D*, the ratio of *k*_*cat*_/*K*_*m*_ for the correct nucleotide divided by *k*_*cat*_/*K*_*m*_ for the mismatch) are shown for each correct incorporation and misincorporation reaction. For ease of comparison, we have grouped the bar graphs by different classes of incorporation: correct base pairs (*green*), Purine/Pyrimidine(T) (*yellow*), Pyrimidine/Purine(T) (*purple*), Purine/Purine(T) (*blue*), Pyrimidine/Pyrimidine(T) (*dark blue*), and identical bases (*red*). The “(T)” next to each class identifies the templating base. *Solid colored bars* indicate parameters that were well constrained by the data while checkered bars only had upper or lower limits on the parameters of interest. Error limits on fitted parameters are listed in Table 2.

All misincorporation reactions, except the wobble mispair U:GTP, follow a 3-step nucleotide incorporation scheme with reversible chemistry followed by slow product release (Fig. 1; Table 1). Our global mechanism-based data fitting yields intrinsic rate constants. To better understand how these rate constants affect the rate and specificity of nucleotide incorporation, we calculated *k*_*cat*_, *K*_*m*_, and *k*_*cat*_/*K*_*m*_ for each base pair using the equations given in the materials and methods section. The results are listed in Table 2 and plotted as bar graphs in Figure 3. For correct incorporations, *k*_*cat*_ was equal to *k*_*2*_ (Table 1) and *K*_*m*_ was similar to 1/*K*_*1*_, but more precisely defined by *K*_*m*_ = (*k*_*-1*_+*k*_*2*_)/*k*_*1*_ as *k*_*-2*_ << *k*_*2*_ and *k*_*3*_ >> *k*_*2*_. Values for *k*_*cat*_/*K*_*m*_ only varied by a factor of ∼12 for correct nucleotide incorporations, generally reflecting the different concentrations of nucleotides in the cell, where more abundant nucleotides had lower *k*_*cat*_/*K*_*m*_ values for correct incorporation.

**Table 2:** Calculated kinetic parameters for all base pair combinations

Templating base	NTP	*k*_*cat*_/*K*_*m*_ (M^-1^s^-1^)	*k*_*cat*_ (s^-1^)	*K*_*m*_ (mM)	Discrimination
A	ATP	[<1.3] x 10^1^	[<1.1] x 10^-2^	0.9 ± 0.4	[>1.6] x 10^5^
	CTP	[1.2 ± 0.8] x 10^1^	[3 ± 2] x 10^-3^	0.3 ± 0.1	[2 ± 1] x 10^5^
	GTP	[<1.5] x 10^-2^	[<4.2] x 10^-5^	3.1 ± 0.7	[>1.4] x 10^8^
	**UTP**^a^**(9)**	[2.3 ± 0.2] x 10^6^	[3.1 ± 0.2] x 10^2^	0.130 ± 0.009	1
C	ATP	[3 ± 2] x 10^1^	[5 ± 3] x 10^-3^	0.15 ± 0.5	[2 ± 1] x 10^5^
	CTP	[<5] x 10^-1^	[<4.7] x 10^-4^	1.4 ± 0.7	[>7] x 10^6^
	**GTP**^a^	[4.7 ± 1.0] x 10^6^	[4.2 ± 0.6] x 10^2^	0.09 ± 0.02	1
	UTP	[<1] x 10^1^	[<9.8] x 10^-4^	0.13 ± 0.04	[>4] x 10^5^
G	ATP	[<7] x 10^-1^	[<6.8] x 10^-4^	1.1 ± 0.5	[>1.3] x 10^7^
	**CTP**^a^	[9.2 ± 0.4] x 10^6^	[6 ± 1] x 10^2^	0.065 ± 0.010	1
	GTP	[2 ± 1] x 10^1^	[2 ± 1] x 10^-3^	0.131 ± 0.050	[5 ± 2] x 10^5^
	UTP	[4 ± 3] x 10^1^	[6 ± 5] x 10^-3^	0.16 ± 0.09	[2 ± 1] x 10^5^
U	**ATP**^a^**(9)**	[7.4 ± 1.6] x 10^5^	[2.4 ± 0.3] x 10^2^	0.32 ± 0.05	1
	CTP	[<3.8] x 10^-3^	[<6.9] x 10^-6^	2.2 ± 0.5	[>1.5] x 10^8^
	GTP	[1 ± 0.7] x 10^2^	[4 ± 2] x 10^-1^	4 ± 2	[7 ± 5] x 10^3^
	UTP	[<13] x 10^1^	[<1.08] x 10^-2^	0.11 ± 0.03	[>5] x 10^3^

a Bases in bold font represent the correct NTP for the given templating base from which the discrimination is calculated from the ratio of k_cat_/K_m_ values as described in the text.

For every misincorporation reaction, *k*_*cat*_ was significantly reduced relative to correct nucleotide incorporation. The fastest *k*_*cat*_ for misincorporation was 0.4 s^-1^ for U:GTP which is 1000-fold slower than *k*_*cat*_ for the correct C:GTP incorporation. Most of the remaining mismatches showed *k*_*cat*_ values ranged from 10^-2^ to 10^-4^s^-1^, although two mismatches were incorporated at rates too slow to measure accurately, A:GTP (<4.2 × 10^-5^s^-1^) and U:CTP (<6.9 × 10^-6^s^-1^). Interestingly, we expected fast misincorporation for the U:GTP wobble mismatch, but the results show some asymmetry in that G:UTP incorporation is 70-fold slower and U:GTP. This may reflect steric effects that would inhibit formation of the wobble mismatch in one direction but not the other (17, 18, 19, 20).

Misincorporation reactions displayed a broad range of *K*_*m*_ values (110 μM to 4 mM), with some comparable to correct nucleotide incorporation and others more than 10-fold weaker than correct base pairs. The low *K*_*m*_ values observed for some misincorporation reactions can be attributed to the slow PP_i_ release mechanism, which allows the favorable equilibrium constant for the chemistry step to be thermodynamically linked to binding, leading to lower *K*_*m*_ values. It is important to note that the *K*_*m*_ values are not equal to 1/*K*_*1*_ which is defined from the nucleotide concentration dependence of the rate of chemistry and does not account for the slow PP_i_ release step.

Finally, to quantify the efficiency and specificity of mismatch incorporation relative to each correct incorporation for each templating base, we calculated *k*_*cat*_/*K*_*m*_ and the discrimination value, defined as *k*_*cat*_/*K*_*m*_ for the correct nucleotide divided by *k*_*cat*_/*K*_*m*_ for the mismatch, for each possible misincorporation. The results are summarized in Table 2 and plotted in Figure 3. For the misincorporation reactions, *k*_*cat*_/*K*_*m*_ varied over a large range (3.8 × 10^-3^M^-1^s^-1^ to 1 × 10^2^M^-1^s^-1^) and all were at least 10^3^M^-1^s^-1^ smaller than *k*_*cat*_/*K*_*m*_ for the least efficient correct incorporation (U:ATP). Discrimination values calculated from these *k*_*cat*_/*K*_*m*_ values are summarized in Table 2 and plotted in Figure 3. The results indicate that there is a wide range of probabilities of misincorporation with certain mismatches being orders of magnitude more likely to occur than other mismatches. These are commonly referred to as “hot spots” or show up as recurring mutation patterns in studies of viral genome evolution, although analysis of viral genomes inherently selects against lethal mutations. Surprisingly, there is quite a bit of variation in the discrimination values for mismatches in the same class and even asymmetric mismatches have quite different values, potentially reflecting different chemistries of tautomerization or wobble base pairing of bases in the incoming nucleotides vs. templating bases.

## Discussion

Understanding the inherent probability of polymerase errors during genome replication is a fundamental process that directly dictates the frequency and spectrum of mutations that occur during replication and impacts the virus’s ability to adapt to environmental pressures, while maintaining sufficient accuracy to replicate the genome. Here, we used pre-steady state kinetic methods to monitor primer extension by the SARS-CoV-2 RdRp. We measured the rates of incorporation on the time scale of a single enzyme turnover to obtain valid estimates of the specificity constant for each base pair combination. In contrast, typical steady state approaches fail to provide accurate measurements for processive enzymes because dissociation of the duplex oligonucleotide from the enzyme is rate-limiting and masks the faster incorporation of correct nucleotides relative to mismatches, thereby greatly underestimating fidelity (21, 22, 23, 24, 25). Furthermore, kinetic analysis of the nucleotide concentration dependence of primer extension using global data fitting allowed us to provide estimates of the apparent ground state nucleotide binding affinity (1/*K*_*1*_), the rate of chemistry (*k*_*2*_), and in most cases, the rate of the reverse of chemistry (*k*_*-2*_) and the rate of PP_i_ release (*k*_*3*_). From these rate constants, we calculated *k*_*cat*_, *K*_*m*_, and *k*_*cat*_/*K*_*m*_ for each possible incorporation reaction. The ratio of *k*_*cat*_/*K*_*m*_ for each mismatched nucleotide relative to that for the cognate base pair gives the probability for misincorporation. By examining all 12 possible mismatches, our analysis provides a complete picture of the selectivity for natural nucleotides by the SARS-CoV-2 RdRp. It is important to acknowledge that our studies were conducted using a single sequence context and there may be smaller differences in discrimination due to sequence-dependent changes in duplex geometry. Our study is intended only to quantify the differences in nucleotide discrimination due solely to changes in base-pair combinations.

Surprisingly, all but one misincorporation reaction catalyzed by the RdRp showed progress curves with a significant dependence of the amplitude of the reaction on the nucleotide concentration (Fig. 2). As shown in prior work (11, 12, 13, 26), this phenomenon is the kinetic signature of a slow PP_i_ release mechanism which allows the chemical reaction to come to an equilibrium that is linked to nucleotide binding. For correct incorporations, PP_i_ release is rapid and largely irreversible, which drives the reaction forward and precludes the reverse of chemistry (*k*_*-2*_ in Fig. 1) from being observed in a single turnover. In contrast, for mismatched incorporations, PP_i_ release is slow. Because this step no longer strongly favors product release, the chemical step can reach equilibrium—meaning that the reverse rate (*k*_*-2*_) becomes measurable as it affects the amplitude of the reaction. Under these conditions, the chemical equilibrium becomes functionally linked to nucleotide binding, leading to the observed nucleotide concentration dependence of the observed amplitude. This was first observed in analysis of the incorporation of 8-oxo-dGTP by the human mitochondrial DNA polymerase (11), but has subsequently been seen for both DNA-dependent DNA polymerases and RNA-dependent RNA polymerases (12, 13, 26, 27). In this prior work, direct measurement established that PP_i_ release was rate-limiting. Slow PP_i_ release may be a conserved mechanism for polymerases to improve selectivity by allowing reversal of incorporation and release of the bound mismatched nucleotide. Studies of Remdesivir incorporation by the SARS-CoV-2 RdRp have shown that it is efficiently incorporated in a manner similar to natural nucleotides (fast PP_i_ release), enabling effective competition with ATP in cells (9). More recently, a slow PP_i_ release mechanism was identified as a key factor contributing to the enhanced antiviral efficacy of 1′-cyanocytidine, as it slows the overall rate of viral replication but is not a chain terminator (28). Future work will determine whether this kinetic framework is broadly applicable to other antiviral nucleoside analogs targeting viral polymerases. MD simulations of the process of PP_i_ release by HIV reverse transcriptase revealed trajectory that involved charged lysine residues along the pathway (29). This result suggests that the PP_i_ release step may involve active participation of residues in the enzyme and could be regulated allosterically.

Another method that could provide a measurement of the reverse rate of chemistry is based on examining the PP_i_ concentration dependence of pyrophosphorolysis (the reverse of the chemical reaction). We published the first application of this method three decades ago (23, 24) and presented the naïve interpretation that the maximum rate of reaction was limited by the rate of reversal of chemistry. However, subsequent analysis has shown that the reaction is not so simple and requires comprehensive global fitting of data for the forward and reverse reactions, measurements of the rates of the ligand-induced conformational changes using an artificial fluorescent amino acid, and estimates for the rates of translocation (25). In addition, the effect of PP_i_ as a competitive inhibitor of NTP binding must also be measured. In the most advanced analysis, the rate constant for reversal of chemistry was 140 s^-1^. However, the simple measurement of the concentration dependence of pyro-phosphorolysis gave estimates of 0.22 s^-1^. This gross underestimate is due to several reversible steps in the reaction with kinetic partitioning of intermediate states leading to slower observed rates, much lower than the intrinsic rate constant. Therefore, one cannot simply monitor the concentration dependence of the pyrophosphorolysis to estimate the rate of reversal of the chemical reaction. Further studies on the SARS CoV-2 RdRp will be necessary to determine whether a substrate-induced conformational change step underlies the observed fidelity.

**Figure 4:**
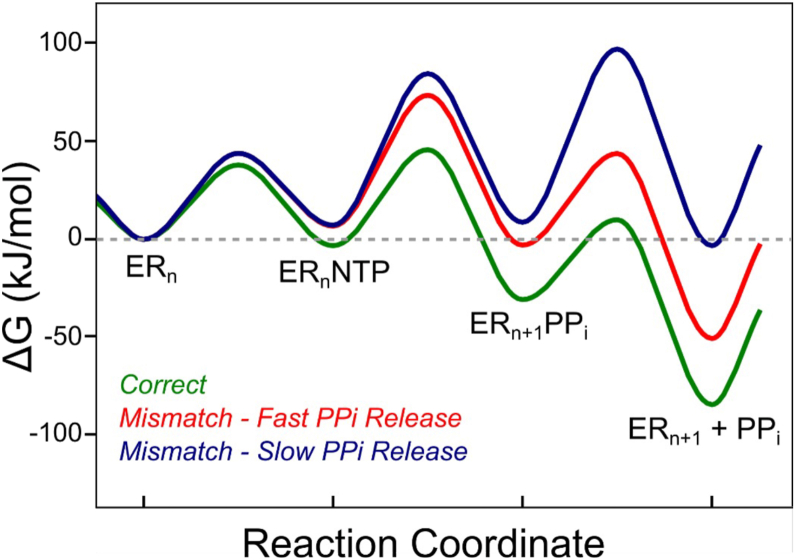
**Free Energy Profile for Nucleotide Incorporation by SARS-CoV-2 Polymerase**. The free energy profile shows the free energy for correct C:GTP incorporation in *green*, U:GTP misincorporation in *red* (fast PP_i_ release), and A:GTP incorporation in *blue* (slow PP_i_ release). The free energy profile was calculated with the rate constants in Table 1 at a temperature of 310 K and with a transmission coefficient of 0.01. The RNA concentration was set to 0.01 μM, the GTP concentration was set to a physiological value of 300 μM (16), and the PPi concentration was set to a physiological value of 1 μM (45).

To quantitatively understand the effect of slow PP_i_ release on specificity, we used the rate constants for three different models of incorporation to plot free energy profiles (Fig. 4). For consistency, all models used in the free energy profile had GTP as the incoming nucleotide while the templating base changed. C:GTP was used as the model for correct nucleotide incorporation (green profile in Fig. 4), U:GTP was used as the model for misincorporation with fast PP_i_ release (red profile in Fig. 4), and A:GTP was used as the model case for misincorporation with slow PP_i_ release (blue profile in Fig. 4). All steps leading up to the highest energy barrier relative to the starting material define *k*_*cat*_/*K*_*m*_. For correct incorporations and misincorporations where PP_i_ release is fast, *k*_*cat*_/*K*_*m*_ is defined by the product of the ground state nucleotide binding affinity and the rate of chemistry, *k*_*cat*_/*K*_*m*_*= K*_*1*_*k*_*2*_. For mismatches with slow PP_i_ release, the highest barrier relative to the starting material is now the PP_i_ release step, so the expression for *k*_*cat*_/*K*_*m*_ now includes ground state nucleotide binding, chemistry, and PP_i_ release, so *k*_*cat*_/*K*_*m*_≈*K*_*1*_*K*_*2*_*k*_*3*_. In addition to introducing another rate-limiting step, slow PP_i_ release also reduces *k*_*cat*_ by allowing for the reversal of chemistry and release of NTP based on the extent to which *k*_*-2*_ is comparable to or greater than *k*_*2*_, *k*_*cat*_=*k*_*2*_*k*_*3*_/(*k*_*2*_+*k*_*-2*_+*k*_*3*_).

While we are not the first to report analysis of fidelity of the SARS-CoV-2 RdRp, previous work used manganese instead of magnesium in steady-state measurements and estimated a fidelity of only 10^-1^ to 10^-3^, corresponding to a discrimination of one error for every 10^1^ to 10^3^ nucleotides incorporated (8). These results are not surprising due to the known mutagenic effect of using manganese as a substitute for magnesium in polymerase reactions (30) and the systematic error introduced by steady state measurements which underestimate discrimination because the rate-limiting enzyme dissociation masks the true rate of correct incorporation. Furthermore, the enzyme activity in the prior study was particularly low, requiring a 1-h incubation to form significant amounts of product. A study using cell culture to measure fidelity of SARS-CoV-2 replication reports an average fidelity of 3.8 × 10^-6^ (31), corresponding to a discrimination of 2.6 × 10^5^. This value is within a factor of 2 of the median discrimination value we report in this study (4.1 × 10^5^). This remarkably close value suggests that the measured *in vitro* polymerase fidelity is sufficient to account for observed fidelity *in vivo*. Therefore, the exonuclease complex may not play a significant role in maintaining genome fidelity *via* proofreading. Since a polymerase-exonuclease complex has not yet been purified and characterized enzymatically, questions regarding the physiological role of the exonuclease remain unanswered.

It is interesting to note that polymerases do not incorporate all mismatches with equal frequency. In the current study discrimination values ranged from 10^3^ to 10^8^, a 5 order-of-magnitude difference in the probabilities for different mismatches. Generally, the most probable misincorporations are U:GTP and U:UTP, followed by C:ATP, A:CTP, G: UTP, and A:ATP. The least probable misincorporations are the A:GTP, G:ATP, U:CTP, and C:CTP. In two cases we observed similar rates of misincorporation when we compared identical base pairs with inverted template *versus* NTP; namely, C: ATP *versus* A: CTP and A:GTP *versus* G:ATP reactions. In contrast we observed asymmetry in the U:GTP *versus* G:UTP, and C:UTP *versus* U:CTP reactions. It has been shown that G and U are able to tautomerize which could affect their ability to form more stable base pairs with mismatched bases (32). The G:U (or dG:dT in DNA) mismatch is known to form a “wobble” base pair that could explain the asymmetry in kinetics observed for U:GTP *versus* G;UTP so that the G:UTP wobble may be more sterically restricted at the active site than U:GTP. This asymmetry was also observed in studies on the human mitochondrial DNA polymerase and the high-fidelity bacteriophage T7 DNA polymerase (17, 18).

Finally, a common misconception about polymerases is the so-called “fidelity-speed trade-off”. This misconception postulates that faster polymerases are less accurate because they have less time to select the correct nucleotide during polymerization or to perform error correction (33, 34, 35). The results presented here directly show that the SARS-CoV-2 polymerase is faster and more accurate that other viral RNA-dependent RNA polymerases and DNA polymerases, contradicting the “fidelity-speed trade-off”. Data show that polymerases gain fidelity by incorporating the correct base pairs at a faster rate relative to a baseline of slow misincorporation. The most well characterized high fidelity enzyme, T7 DNA polymerase, uses a substrate-induced-fit mechanism to rapidly select and incorporate correct base pairs while rejecting mismatches (6, 7, 18, 25). HIV reverse transcriptase has moderate fidelity and is much slower than T7 DNA pol but still uses an induced-fit mechanism (36, 37). Repair enzymes, such as Klenow fragment (21, 22) and Pol β (36, 38, 39, 40) are much slower and have low fidelity. Although Pol β exhibits a two-step nucleotide binding pathway, unlike HIV reverse transcriptase and T7 DNA polymerase, the conformational changes are rapidly reversible and are not a primary determinant of fidelity. Another well studied RNA-dependent RNA polymerase, from Hepatitis C virus, is considerably slower and less accurate than the SARS-CoV-2 polymerase (12, 41, 42). Future work will determine whether the coronavirus RdRp follows an induced-fit mechanism, although our current data indicate that it is both fast and accurate.

While this study provides important insights into the mechanism of nucleotide selectivity for the SARS-CoV-2 RdRp, many important questions remain. For example, does the specificity of the exonuclease complex change when associated with the RdRp to specifically remove mis-incorporated bases during genome replication, or does this complex primarily serve an auxiliary role in genome maintenance and recombination? The exonuclease complex can efficiently remove most nucleoside analog drugs once incorporated, so new analogs that evade the exonuclease could be more effective therapeutically. Addressing these questions will be critical for understanding coronavirus biology as well as designing novel classes of nucleoside analogs with improved antiviral efficacy against COVID-19.

## Experimental procedure

### Enzymes

Tag-free SARS-CoV-2 NSP12,7,8 complex and individual NSP8 were expressed in *Escherichia coli* and purified as previously described (9, 43). Briefly, NSP8 was expressed in BL21*E. coli* harboring the plasmid pcI^ts^,ind^+^-(NSP8) (Addgene Plasmid #160656). Cells were grown in Terrific Broth with ampicillin for selection. Protein was expressed for 3 h by shifting the culture temperature from 30 °C to 42 °C for 20 min, followed by 3 h at 38 °C. The cells were lysed by sonication, and then the lysate was clarified by centrifugation and separated on the following columns: Q Sepharose-FF, SP Sepharose-FF, HiTrap Blue-FF, Superdex 200. The protein was then dialyzed into Storage Buffer-1 (50 mM Tris-HCl pH 7.5, 0.1 mM EDTA, 20 mM KCl, 1 mM DTT, 50% [v/v] glycerol), aliquoted, and stored at −80 °C. Protein concentration was determined by absorbance at 280 nm using the extinction coefficient ε_280_= 19,940 M^-1^cm^-1^ based on the amino acid sequence of NSP8.

The three polypeptides for the NSP12/7/8 complex were co-expressed in BL21*E. coli* harboring the plasmids pG-Tf2 (Takara Bio) and pQE-(NSP12)-pcI^ts^,ind^+^-(NSP7-NSP8) (Addgene Plasmid #160540) (9, 43). Cells were grown in Terrific Broth with kanamycin and chloramphenicol for selection at 30 °C. Protein was expressed overnight at 16 °C after addition of tetracycline, IPTG, and nalidixic acid. Harvested cells were lysed by sonication, then the lysate was clarified by centrifugation, and the NSP12/7/8 complex was purified using the following columns: Q Sepharose-FF, HiTrap Blue-FF, Heparin Sepharose 6-FF, SP Sepharose-FF, Superdex 200. The protein was then dialyzed into Storage Buffer-2 (50 mM Tris-HCl pH 7.5, 0.1 mM EDTA, 50 mM NaCl, 1 mM DTT, 50% [v/v] glycerol), aliquoted, and stored at −80 °C. Protein concentration was determined by absorbance at 280 nm using the extinction coefficient ε_280_= 181,300 M^-1^cm^-1^ for the complex, assuming 1 molecule of NSP12 and NSP7 and two molecules of NSP8 per complex.

### Oligonucleotides

RNA Oligonucleotides in Table 3 were synthesized by Integrated DNA Technologies with RNase-free HPLC purification. Oligos were resuspended in Annealing Buffer (10 mM Tris-HCl pH 7, 50 mM NaCl, 0.1 mM EDTA) and the concentration was determined by absorbance at 260 nm using the extinction coefficients listed in Table 3. FAM-RNA duplex oligonucleotides were annealed by mixing primer and template at a 1:1 M ratio, heating to 75 °C, and cooling slowly to room temperature over approximately 1 h. Oligonucleotides were stored at −20 °C.

**Table 3:** Oligonucleotides used in this study

Oligo name^a^	Oligo sequence (5′ -> 3′)	Extinction Coefficient, 260 nm (M^-1^cm^-1^)
FAM-LS2	[6-FAM]—GUCAUUCUCCUAAGAAGCUA	221,760
LS1.2-A	CUAUCCCCAUGUGAUACGCAUAGCUUCUUAGGAGAAUGAC	397,900
LS1.2-C	CUAUCCCCAUGUGAUACGACUAGCUUCUUAGGAGAAUGAC	397,900
LS1.2-G	CUAUCCCCAUGUGAUACGAGUAGCUUCUUAGGAGAAUGAC	402,600
LS1.2-U	CUAUCCCCAUGUGAUACGAUUAGCUUCUUAGGAGAAUGAC	401,600

a In the text, FAM-RNA refers to an RNA duplex with a 5′-fluoresceine labeled “FAM-LS2” primer with one of the four template strands LS1.2-A, -C, -G, or -U to designate different templating bases for single nucleotide incorporation studies.

### Kinetic experiments

Kinetics experiments were performed in reaction buffer (40 mM Tris-HCl pH 7, 50 mM NaCl, 5 mM MgCl_2_, 1 mM DTT) at 37 °C. A solution of enzyme and RNA was prepared at 2x final concentration in reaction buffer and allowed to equilibrate on ice for 30 min and them warmed to 37 °C before adding the NTP of interest to start the reaction. NTP solutions were purchased from New England Biolabs and prepared as Mg^2+^-NTP to provide a constant free magnesium concentration in the reactions. Time points were quenched by adding EDTA to 0.3 M. Time points for misincorporation reactions were collected by hand mixing to start and stop the reaction. Time points for correct C:GTP and G:CTP were collected on a RQF-3 quench flow instrument (KinTek Corporation). Samples quenched at various times were analyzed by capillary electrophoresis on an ABI 3130xl Genetic Analyzer, equipped with a 36 cm capillary array and nanoPOP-6 polymer (Molecular Cloning Laboratories) at 65 °C using established methods (44). Samples were prepared for analysis by mixing 1 μl of sample with 10 μl of HiDi formamide (Thermo Fisher Scientific). Samples were injected for 60 s at 5 kV. Peak areas were determined with GeneMapper software and the concentration of each product was calculated by dividing the peak area for the product of interest by the total peak area and multiplying this value by the total concentration of RNA used in the reaction. Representative electropherograms for correct and misincorporation are shown in Figure S1. Concentrations given in the text are final concentrations after mixing. Technical replicates: all experiments were performed on at least 2 separate occasions to ensure reproducibility. An experiment in this context is defined as a complete time course for a combination of templating nucleobase/incoming nucleotide at multiple incoming nucleotide concentrations. Biological replicates: two batches of SARS-CoV-2 RdRp were used to collect the experimental data in this manuscript with similar results from each batch.

### Analysis of kinetic data

Data fitting and analysis were performed with KinTek Explorer simulation and data fitting software (14) v11 (www.kintekexplorer.com). This software was also used to prepare figures for kinetic data and the free energy profile in Figure 4. Bar graphs shown in Figure 3 were created in Microsoft Excel. For more information the advantages and methods of global data fitting, see (10).

The model below was entered into KinTek Explorer. Steps 1 to 3 correspond to steps 1 to 3 in the kinetic scheme in Figure 1. Steps 4 and 5 correspond to RNA binding/dissociation steps to the 20 nt long RNA and 21 nt long RNA, respectively. Starting conditions were entered into the software exactly as the experiments were performed and for all experiments the observables contained all species with n + 1 RNA (ER_21_PP_i_+ ER_21_+ R_21_). Data were fit to this model to derive the rate constants in Table 1. To estimate the ground state nucleotide binding constant, *k*_*1*_ was locked at 100 μM^-1^s^-1^. The parameter *k*_*-3*_ was locked at 0 μM^-1^s^-1^. The software was then used to fit the value for *k*_*-1*_ and the ratio of the two numbers, *K*_*1*_, is reported in Table 1. The RNA off rates (*k*_*-4*_ and *k*_*-5*_) were linked and locked at the off rate previously measured (9) and the RNA on rates (*k*_*4*_ and *k*_*5*_) were linked and allowed to float to fit the amplitude observed for all misincorporation reactions for a particular templating base. In the simulation, the enzyme and RNA were allowed to equilibrate for 30 min before starting the reaction by NTP addition to mimic the experimental conditions. Modeling included all steps in the reaction shown below. The syntax for entering mechanisms in KinTek Explorer includes an equal sign representing a reversible reaction. The length of the RNA (R) primer is denoted by the subscript.

Equation 1 $${\text{ER}}_{20}+\text{NTP}={\text{ER}}_{20}N$$

Equation 2 $${\text{ER}}_{20}N={\text{ER}}_{21}{\text{PP}}_{i}$$

Equation 3 $${\text{ER}}_{21}{\text{PP}}_{i}={\text{ER}}_{21}+{\text{PP}}_{i}$$

Equation 4 $$E+R_{20}={\text{ER}}_{20}$$

Equation 5 $$E+R_{21}={\text{ER}}_{21}$$

To estimate the 95% confidence intervals for each parameter, we used the FitSpace (15) function of KinTek Explorer to compute confidence contours. Based on the number of data points and parameters, a chi^2^ threshold of 0.8 was used to estimate the 95% confidence interval (10). For parameters with only an upper or lower limit, we report only these limits Table 2. In the final analysis we also provide estimates of the standard deviation of data based on the residuals in the globally fit progress curves. This analysis provides a robust estimate of the random errors in data collection based on a minimal model based on few variable parameters. Each experiment was performed at least twice.

Kinetic parameters *k*_*cat*_, *K*_*m*_, and *k*_*cat*_/*K*_*m*_ were calculated from estimated rate constants using the equations below for a 3-step incorporation model (including PP_i_ release). Our data provide estimates for *K*_*1*_, but not the individual rate constants, *k*_*1*_ and k_-1_. For these calculations, we locked *k*_*1*_ at a conservative estimate of the diffusion-limited rate for nucleotide binding (100 μM^-1^s^-1^) and fit to derive *k*_*-1*_ which was then used to calculate *K*_*1*_.

kcat=k2k3k2+k−2+k3Km=k2k3+k−1(k−2+k3)k1(k2+k−2+k3)kcatKm=k1k2k3k2k3+k−1(k−2+k3)

Discrimination values were calculated by dividing *k*_*cat*_/*K*_*m*_ for the correct nucleotide incorporation at a specific templating base by *k*_*cat*_/*K*_*m*_ for the mismatch of interest.

## Data availability

All data are available in the supporting information in the form of KinTek Explorer mechanism files, https://doi.org/10.5281/zenodo.16999782. These files can be opened (without needing a license) using KinTek Explorer software available at https://kintekexplorer.com, where video tutorials are also available to illustrate use of the software. The mechanism files also show how the data were modeled and users can explore alternative models (10).

## Supporting information

This article contains supporting information.

## Conflict of interest

The authors declare that they have no conflicts of interest with the contents of this article.
