# Supplementary material for: Slow PPi release enhances fidelity of the SARS-CoV-2 RNA dependent RNA polymerase[image]
Source: J Biol Chem. 2026 Apr 17;302(6):111457. doi: 10.1016/j.jbc.2026.111457 (PMC13208697; doi:10.1016/j.jbc.2026.111457)
Supplement: Supplementary material [file mmc1.docx]

**Slow PP_i_ release enhances fidelity of the SARS-CoV-2 RNA dependent RNA polymerase**

Tyler L. Dangerfield^1,2^, Ingrid Marko^1^, Kenneth A. Johnson^1^*

**Supporting Information**

| **Table S1: Nucleotide Concentrations Used in Experiments in Figure 1** | | | | | |
| --- | --- | --- | --- | --- | --- |
| Templating Base | Nucleotide | Concentrations used (μM) | Templating Base | Nucleotide | Concentrations Used (μM) |
| A | ATP | 200,500,1100,2000 | G | ATP | 600,1000,2000,3000 |
|  | CTP | 100,250,1000,2000 |  | CTP | 1.25,2.5,5,10,15,  25,50,100 |
|  | GTP | 1000,2000,3000,  4000 |  | GTP | 1000,2000,3000 |
|  | UTP | 2.5,5,10,20,40,80,  150 |  | UTP | 250,500,1000,2000 |
| C | ATP | 90,275,500,1000 | U | ATP | 5,10,20,40,80,150,  225,400 |
|  | CTP | 500,1000,2000,3000 |  | CTP | 500,1000,2000,3000 |
|  | GTP | 19,30,48,77,107,  150,210 |  | GTP | 250,500,1000,2000 |
|  | UTP | 90,250,500,1000 |  | UTP | 225,525,1000,2000 |


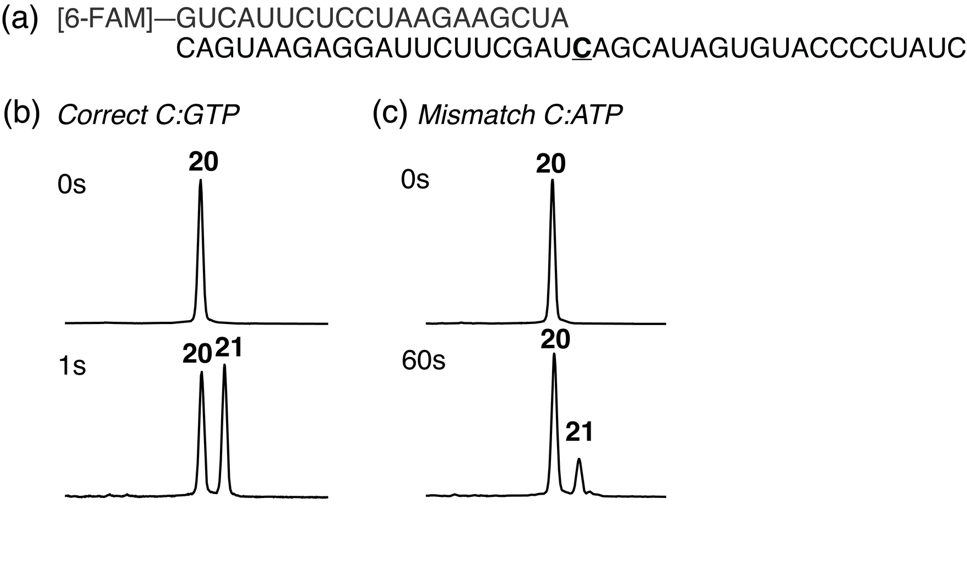


Figure S1: Representative electropherograms of correct and mismatched nucleotide incorporation by the NSP12/7/8 complex. (a) Double-stranded RNA substrate containing a 5′ FAM label on the primer strand to enable fluorescence detection. The templating base corresponding to the incoming nucleotide is indicated in bold. Shown are representative single time point traces to illustrate the detection and quantification of RNA products. (b) Correct incorporation of GTP opposite a templating cytidine at 100 µM GTP (1 s reaction). (c) Misincorporation of ATP opposite a templating cytidine at 100 µM ATP.
